# Supplementary material for: A low rate of end-stage kidney disease in membranous nephropathy: A single centre study over 2 decades
Source: PLoS One. 2022 Oct 13;17(10):e0276053. doi: 10.1371/journal.pone.0276053 (PMC9560622; doi:10.1371/journal.pone.0276053)
Supplement: S1 Table — (DOCX) [file pone.0276053.s001.docx]

| **Variable** | **Mild Proteinuria (<350, g/mol) n=35*** | **Moderate Proteinuria (350-800, g/mol) n=72*** | **Severe Proteinuria (>800, g/mol) n=66*** | **P value**  **(for mild vs severe proteinuria)** |
| --- | --- | --- | --- | --- |
| Age at time of biopsy (years) | 53 (37-68) | 58 (46-68) | 60 (48-67) | 0.377 |
| Male, *n* (%) | 14 (40) | 25 (35) | 24 (36) | 0.812 |
| \|  \| \| --- \|   Caucasian, *n* (%) | 33 (94) | 65 (90) | 55 (83) | 0.134 |
| \|  \| \| --- \|   Diabetes *n* (%) | 0 (0) | 7 (10) | 12 (18) | **0.008** |
| Hypertension^,^ *n* (%) | 21 (60) | 39 (54) | 35 (53) | 0.524 |
| Cardiovascular disease, *n* (%) | 5 (14) | 11 (15) | 12 (18) | 0.683 |
| Systolic BP, mmHg | 132 (118-140) | 137 (126-148) | 135 (122-153) | 0.305 |
| Diastolic BP, mmHg | 78 (69-90) | 79 (70-85) | 79 (70-88) | 0.706 |
| Haemoglobin (g/L) | 127 (114-135) | 131 (120-145) | 132 (119-143) | 0.216 |
| Albumin (g/L) | 34 (28-39) | 27 (22-32) | 26 (20-28) | **<0.001** |
| Corrected Calcium (mmol/L) | 2.35 (2.22-2.43) | 2.39 (2.29-2.48) | 2.39 (2.31-2.48) | 0.147 |
| Phosphate (mmol/L) | 1.19 (1.04-1.40) | 1.26 (1.04-1.32) | 1.18 (1.04-1.39) | 0.949 |
| anti-PLA2R positive, *n* (%) | 4 (11) | 13 (18) | 17 (26) | 0.865 |
| anti-PLA2R (U/ml) | 66 (17-132) | 164 (39-474) | 63 (16-158) | 0.816 |
| eGFR (mls/min/1.73 m^2^) | 73 (50-90) | 75 (56-90) | 78 (46-90) | 0.845 |
| Creatinine (µmol/L) | 88 (62-123) | 92 (70-120) | 86 (71-140) | 0.895 |
| Remission, *n* (%) | 30 (86) | 59 (82) | 43 (65) | **0.015** |
| Relapse, *n* (%) | 14 (40) | 24 (33) | 19 (29) | 0.571 |
| Received ACEi/ARB, *n (%)* | 34 (97) | 68 (94) | 62 (94) | 0.507 |
| Received immunosuppression, *n* (%) | 12 (34) | 34 (47) | 48 (73) | **<0.001** |
| Progression to RRT, *n* (%) | 5 (14) | 3 (4) | 9 (14) | 0.790 |
| Death, *n* (%) | 9 (26) | 22 (31) | 22 (33) | 0.508 |
| Follow up (months) | 68 (36-112) | 65 (28-101) | 46 (27-94) | 0.203 |

*Proteinuria data not available for 5 patients

Continuous variables presented as median (interquartile range), p-value by Mann-Whitney U test. Categorical values presented as number (percentage), p-value by Chi-squared test.

ACEi, angiotensin converting enzyme inhibitor; ARB, angiotensin receptor blocker; DBP, diastolic blood pressure; eGFR, estimated glomerular filtration rate; MN, membranous nephropathy; anti-PLA2R, anti-phospholipase 2A receptor; RRT, renal replacement therapy; SBP, systolic blood pressure; uPCR, urine protein creatinine ratio.
