# Supplementary material for: A low rate of end-stage kidney disease in membranous nephropathy: A single centre study over 2 decades
Source: PLoS One. 2022 Oct 13;17(10):e0276053. doi: 10.1371/journal.pone.0276053 (PMC9560622; doi:10.1371/journal.pone.0276053)
Supplement: S2 Table — (DOCX) [file pone.0276053.s002.docx]

| **ariable** | **2000-2011 (n=85)** | **2012-2019 (n=93)** | **P value** |
| --- | --- | --- | --- |
| Age at time of biopsy (years) | 56.3 (39.7-64.6) | 61.8 (48.1-70.3) | **0.014** |
| Male, *n* (%) | 54 (63.5) | 59 (63.4) | 0.990 |
| Caucasian, *n* (%) | 77 (90.6) | 81 (87.1) | 0.461 |
| Diabetes *n* (%) | 8 (9.4) | 11 (11.8) | 0.602 |
| Hypertension, *n* (%) | 51 (60.7) | 46 (49.5) | 0.133 |
| Cardiovascular disease, *n* (%) | 14 (16.5) | 14 (15.1) | 0.795 |
| Systolic BP, mmHg | 133 (120-147) | 136 (125-149.5) | 0.421 |
| Diastolic BP, mmHg | 76 (69.5-88.0) | 80 (70-85) | 0.307 |
| Haemoglobin (g/L) | 129 (115.5-144) | 131 (120-141.5) | 0.702 |
| Albumin (g/L) | 26 (21.5-34) | 28 (23.5-32) | 0.664 |
| Corrected Calcium (mmol/L) | 2.28 (2.20-2.38) | 2.44 (2.38-2.52) | **<0.001** |
| Phosphate (mmol/L) | 1.27 (1.07-1.44) | 1.18 (1.03-1.32) | 0.076 |
| eGFR (mls/min/1.73 m^2^) | 66.9 (47.5-90.0) | 80.9 (57.3- 90.0) | **0.039** |
| Creatinine (µmol/L) | 96 (77.5-136.5) | 81 (64.5-117.5) | **0.006** |
| uPCR (g/mol) | 598 (345.5-978) | 750 (430.5-1110) | 0.068 |
| Remission, *n* (%) | 65 (79.3) | 69 (75.8) | 0.588 |
| Relapse, *n* (%) | 29 (45.3) | 28 (41.8) | 0.684 |
| Received ACEi/ARB, *n (%)* | 78 (94.0) | 89 (95.7) | 0.604 |
| Received immunosuppression, *n* (%) | 38 (45.8) | 56 (60.9) | **0.046** |
| Progression to RRT, *n* (%) | 11 (13.3) | 7 (7.6) | 0.261 |
| Death, *n* (%) | 37 (43.5) | 16 (17.2) | **<0.001** |
| Follow up (months) | 95 (37.5-145) | 44 (24-79.5) | **<0.001** |

Continuous variables presented as median (interquartile range), p-value by Mann-Whitney U test. Categorical values presented as number (percentage), p-value by Chi-squared test.

ACEi, angiotensin converting enzyme inhibitor; ARB, angiotensin receptor blocker; DBP, diastolic blood pressure; eGFR, estimated glomerular filtration rate; MN, membranous nephropathy; anti-PLA2R, anti-phospholipase 2A receptor; RRT, renal replacement therapy; SBP, systolic blood pressure; uPCR, urine protein creatinine ratio.
