# Supplementary material for: A low rate of end-stage kidney disease in membranous nephropathy: A single centre study over 2 decades
Source: PLoS One. 2022 Oct 13;17(10):e0276053. doi: 10.1371/journal.pone.0276053 (PMC9560622; doi:10.1371/journal.pone.0276053)
Supplement: Attachment — Submitted filename: Response to reviewers.docx [file pone.0276053.s004.docx]

Dear Academic Editor,

In response to your points:

1. The manuscript meets PLOS ONE’s style requirements.
2. We have added the required ‘competing interests’ statement to the cover letter as requested.

Dear reviewers,

Many thanks for all of your comments. Our responses are detailed below.

Reviewer 1

Given the growing evidence for SGLT2 inhibitors as a means of supportive care in CKD, consideration about using SGLT2 inhibitors may be added in the discussion.

This has been commented on in the discussion.

Reviewer 2

Although they used the proportion of death or progression to RRT as markers for outcomes of interest, these parameters are affected by the observational period. For example, the follow-up periods of the patients without remission was clearly shorter than those of the patients with partial or complete remission in Table 4.

Therefore, the proportion of death or progression to RRT could be underestimated in the patients with former group. I recommend using times per patient-month or patient-year as a marker for outcome.

The reason for the follow up period being shorter amongst those who do not achieve remission is because the end date for follow up period includes progression to RRT and death. If they have more of these events then it follows that the follow up duration will be less. The proportion who progressed to RRT or died will not be underestimated as these parameters were assessed in all patients. As such, whilst we recognise the utility of per patient-month/ per patient-year, we have not switched to this for our analysis.

Explanatory variables of Table 2 to 5 should be limited to baseline characteristics. It is strange that they compared the proportion of relapse between patients without remission, those with partial remission and those with complete remission in Table 4.

Variables assessed in table 2 are all baseline variables, consistent with performing Cox-regression analysis. Variables in tables 3-5 include baseline and outcome measures such as such as progression to RRT and death as these outcomes are a key part of the study.

We have removed the comparison of relapse rates in table 4.

Although the title was a low rate of ESKD in MN, they tried to identify the risk factors for death or ESKD among patients with MN. At least, they should cite previous reports showing the rate of ESKD in MN.

Chembo et al. reported that less than 20% of patients with MN had reached ESKD at 10 years follow up using data obtained from the New Zealand Glomerulonephritis Study [Chembo CL et al. Nephrology (Carlton) 20:899-907,2015].

Recently, Hamano et al. reported that incidence of dialysis among patients with MN was approximately 30/1000 person-years, and the risk was 0.45 times lower than patients with IgA nephropathy using the CKD-JAC Study, a multicenter, prospective observational cohort study in Japan. [Hamano T et al, Nephrol Dial Transplant. 2022 Mar 22:gfac134. Online ahead of print].

Many thanks for highlighting these, we have referenced them in the discussion.

To investigate the clinical efficacy of the modified Ponticelli immunosuppressive regime, the control group should be patients without immunosuppression.

The reason that we used the control group of ‘other immunosuppression’ was because we have already used a control group of ‘no immunosuppression’ in table 1. Ideally we would have liked to compare individual specific types of immunosuppression, but as commented in the paper, this was not possible due to the low numbers in each of the different categories (see figure 2 for a full breakdown of these).

In the Conclusion, the authors only repeated to present the results.

We have amended the conclusion to provide more analysis.

Reviewer 3

1. The authors showed that 33.7% of patients reached spontaneous complete or partial remission, and 41.6% of patients reached remission by immunosuppressive treatment. In case of spontaneous remission, how long do you observe the patients without immunosuppression treatment? What is the inducement of the initiation of immunosuppression therapy in the patients with MN?

We generally observe patients for 6 months with supportive treatment before considering immunosuppression treatment. However, we may decide to start immunosuppression treatment earlier depending on the clinical circumstances.

We tend to start induction immunosuppression with either Modified Ponticelli or a calcineurin inhibitor for the majority of patients, with a decision usually made at our multi-disciplinary meeting involving nephrologists and pharmacists.

2. The patients are enrolled from 2000 until 2019. During two decades of study period, therapy for PMN was changed substantially. Could the authors compare the remission rate between early period from 2000 to 2010 and late period from 2011 to 2019?

We have run this analysis and included it as supplementary table 2. There was no significant difference in remission (79.3 % in earlier group vs 75.8% in later group, p=0.59) or relapse (45.3% vs 41.8%, p=0.68) rates between the two time periods. More immunosuppression was used in the later period group (60.9% vs 45.8%, p=0.046).

3. Please show the Kaplan-Mayer analysis for cumulative probabilities of remission against follow-up period.

Figure 3 depicts Kaplan-Meier curves for both overall survival and freedom from RRT for those patients who achieved no, partial or complete remission. If there is further Kaplan-Meier analysis that you were wanting to see then let us know.

4. Deaths was reported in 53 cases out of 178 patients. Approximately 30% of patients died. It is very high if the mortality was caused by kidney diseases. Please identify the cause of death.

As the study period was over 20 years with a median age at presentation of 58.3 years, the mortality data will include non-kidney related death. Unfortunately we were not able to collect specific cause of death data due to the retrospective nature of the study.

Reviewer 4

1. With respect to the ratio of remission or RRT, the ratio is not appropriate, but should be presented as the incidence of RRT with a unit of /patient/yr.

We have kept the rate of remission, relapse, progression to RRT and death as a percentage (number of outcomes observed within the group being described) as we believe this accurately describes these outcomes for the total cohort and various subgroup analyses.

2. To define the partial and complete remission of proteinuria, please put the appropriate references.

Reference has been included in the appropriate section of the methods section.

3. Please perform the subgroup analysis after blood anti-PLA2R antibody level was started to be assessed routinely even if “our anti-PLA2R data is far from complete”. Or, please specify how incomplete they are. That topic is what not a few nephrologists are so interested in.

We understand the importance of anti PLA2R testing for all patients with membranous nephropathy. Unfortunately we only had 63 anti-PLA2R results available, and of these 34 were positive. As such any subgroup analysis will be difficult to extrapolate to a wider population due to the low numbers involved. Furthermore, when the test was first available only a positive/ negative result was available, but more recently a titre result has been reported, further complicating potential analysis. A prospective study would be a very interesting next step to address these issues.

Minor concerns:

In the abstract MN has been spelled out and the median observation period has been specified.

In the Abstract, the referee does not understand “Amongst the whole cohort, those who went into remission did better than those who did not.”

This has been clarified in the abstract.

Also, without the definition of high- or low-risk, it is difficult to understand “Those classified as high-risk also had worse outcomes than those at low-risk”.

This has been clarified in the abstract. Note that we included the reference for the classification in the results section (KDIGO 2021 Clinical Practice Guideline for the Management of Glomerular Diseases. Kidney Int. 2021;100:S1-s276.).
